# Improved Multiscale Entropy Technique with Nearest-Neighbor Moving-Average Kernel for Nonlinear and Nonstationary Short-Time Biomedical Signal Analysis

**DOI:** 10.1155/2018/8632436

**Published:** 2018-03-05

**Authors:** S. P. Arunachalam, S. Kapa, S. K. Mulpuru, P. A. Friedman, E. G. Tolkacheva

**Affiliations:** ^1^Department of Biomedical Engineering, University of Minnesota, Minneapolis, MN, USA; ^2^Department of Cardiovascular Medicine, Mayo Clinic, Rochester, MN, USA; ^3^Department of Cardiovascular Medicine, Mayo Clinic, Phoenix, AZ, USA

## Abstract

Analysis of biomedical signals can yield invaluable information for prognosis, diagnosis, therapy evaluation, risk assessment, and disease prevention which is often recorded as short time series data that challenges existing complexity classification algorithms such as Shannon entropy (SE) and other techniques. The purpose of this study was to improve previously developed multiscale entropy (MSE) technique by incorporating nearest-neighbor moving-average kernel, which can be used for analysis of nonlinear and non-stationary short time series physiological data. The approach was tested for robustness with respect to noise analysis using simulated sinusoidal and ECG waveforms. Feasibility of MSE to discriminate between normal sinus rhythm (NSR) and atrial fibrillation (AF) was tested on a single-lead ECG. In addition, the MSE algorithm was applied to identify pivot points of rotors that were induced in *ex vivo* isolated rabbit hearts. The improved MSE technique robustly estimated the complexity of the signal compared to that of SE with various noises, discriminated NSR and AF on single-lead ECG, and precisely identified the pivot points of *ex vivo* rotors by providing better contrast between the rotor core and the peripheral region. The improved MSE technique can provide efficient complexity analysis of variety of nonlinear and nonstationary short-time biomedical signals.

## 1. Introduction

Biomedical signals are characteristic of their corresponding physiological events and carry specific signatures [1]. Consequently, deciphering signal characteristics provides information regarding underlying processes that can be useful to inform or guide therapy. Most physiological processes are characterized by specific signals that reflect the nature and activities of such processes, which can contain biochemical, electrical, or physical information coming from molecular, cellular, organ, or systemic level sources [2]. Hence in a disease state, alterations to these physiological processes yield signal signatures that are different in some aspects from the normal state [1]. Electrocardiogram (ECG), electroencephalogram (EEG), electromyogram (EMG), electroretinogram, and so on are some examples of electrical signals that are commonly acquired for risk assessment, prognosis, diagnosis, therapy evaluation, and prevention of various diseases [3].

Biomedical signal analysis requires accurate quantification of the system state to distinguish between normal and pathological function or to predict the future state of the system using only short time series data that may only last a few seconds. Signal analysis is typically complicated by contamination with electromagnetic interference, power line interference, zero mean white noise, pink noise, brown noise from electrode movement, and other random noise [2].

Many biomedical signals are captured only for 3–8 s and therefore are short nonstationary and/or nonlinear time series data, which prevent ordinary biomedical analysis algorithms from completely capturing their intrinsic complexity. For instance, Shannon entropy (SE) is commonly used for biomedical complexity analysis of EEG and ECG recordings [3–5]. However, one of the major limitations of the SE approach is related to the specific characteristics of the nonstationary and/or nonlinear time series data that work well for long but is not robust for short data segments. Several other symbolic dynamic approaches that use various entropy-based measures, such as Kolmogorov entropy, spectral entropy, wavelet entropy, permutation entropy, approximate entropy, and sample entropy, have been proposed to capture the intrinsic dynamics of nonstationary time series data to quantify their complexity [6–12]. However, it has been shown that these various entropy-based methods are efficient only for long time series and do not completely capture the complexities of shorter nonstationary time series data [13].

Recently, a multiscale entropy (MSE) technique was proposed for coarse-grained time-scaling procedures to offer more robust determination of the complexity of time series data [14]. Such coarse-graining procedures may result in invalid entropy value estimation for shorter time series; and this limitation was addressed by implementing a moving-average time series estimate [15]. However, the moving average in prior work was only performed in the forward direction, which can lead to significant underestimation of the complexity information that is present in the time series data [15]. Several variants of MSE have thus been proposed [16], but all of them provide only slight modifications from the original technique [15] and specifically depend on a one-sided moving average, which yields biased entropy estimates over different time scales. Several variants of MSE have been applied to test synthetic biomedical datasets without a rigorous demonstration of their feasibility for a biomedical application [17–19]. Therefore, using entropy-based techniques for rigorous complexity analysis of a biomedical signal in normal and diseased states has been very limited. Several researchers have used MSE technique for a variety of analysis using cardiac signal analysis [20–25] showing some promise for complexity assessment to aid diagnosis. However, the authors identify a major limitation of these MSE variants with the systematic bias in the one-sided average which may have affected the results. The introduced bias becomes extremely important to consider for improvement because most biological signals embed only subtle changes in short time series data which may have significant diagnostic potential that could be lost with such bias.

The challenge with short time series data analysis comes from the fact that the complexity of the data may not embed in the raw signal. Previously developed MSE techniques were introduced with time-averaged time series over multiple time scales for short time series analysis [15]. However, forward averaging introduces a systematic bias in the complexity estimation. To overcome this limitation, we proposed a nearest-neighbor moving-average kernel to better capture the complexity of nonlinear, nonstationary short time series data. We introduce the concept of “memory” by taking into account the past and future time series value while computing the nearest-neighbor moving average for time series data. Therefore, we introduce the time-scale factor “*τ*”, which represents time scaling in both forward and reverse directions with respect to a particular time point. Once this new time series is derived, the MSE estimate can be obtained by calculating the entropy of the new time series sample over multiple time scales to fully capture the intrinsic complexity of nonlinear and nonstationary time series data.

In this work, we propose an improved MSE technique, which includes significant and robust modification of the previously described MSE techniques. Specifically, we propose computation of the new time series with a nearest-neighbor moving-average kernel that uses information from the “past” and “future” values to accurately capture the intrinsic dynamics of the short time series. Our modification will allow a robust analysis of nonlinear and nonstationary time series.

The efficacy and robustness of the improved MSE technique will be validated by performing noise analysis with respect to white, pink, and brown noise, which are commonly present in cardiac signals such as the ECG. Since SE has been used widely for biomedical signal complexity analysis so far, we will use it as a “gold standard”, and we will compare the performance of the novel MSE technique with SE. We further hypothesized that the improved MSE technique will robustly quantify the complexity of nonlinear and nonstationary short time series data. We tested this hypothesis by applying the improved MSE technique for the analysis of the two physiological applications: (i) discrimination between normal sinus rhythm (NSR) and atrial fibrillation (AF) using a single-lead ECG and (ii) the accurate identification of the pivot point of rotors, which are potential ablation targets for AF and other arrhythmias.

## 2. An Improved MSE Technique with Nearest-Neighbor Moving-Average Kernel

The improved MSE algorithm consists of several steps as described below. Let *x* = {*x*_1_, *x*_2_, *x*_3_,…, *x*_*N*_} represent the electrogram time series of length *N*.

**Figure 1:**
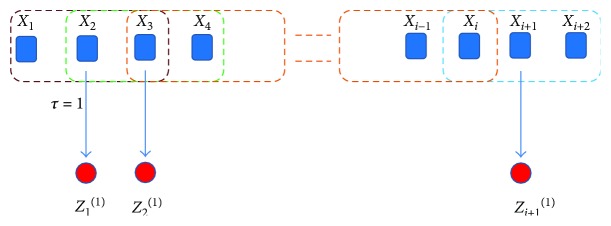
Schematic illustration to produce nearest neighbor moving-average time series with scale factor *τ* = 1 for the MSE algorithm. Blue squares represent raw time series data, and red dots represent the nearest-neighbor moving-averaged time series from which MSE is obtained. Brown squares represent the moving-window-averaging kernel for the raw second time point (*X*_2_) that averages one neighbor on both sides with *τ* = 1 to produce the first new time series point *Z*_1_^(1)^. Similarly, green square produces *Z*_2_^(1)^ and so on (orange square) with the blue square producing the last time series point *Z*_*i*+1_^(1)^.

(1) Nearest-neighbor moving-averaged time series *z^τ^* is computed for the chosen time-scale factor “*τ*” as illustrated in Figure 1 using the following equation:
  (1) $$\begin{array}{c} z_{j}^{\tau }=\frac{1}{\left(2\tau +1\right)}\overset{2\tau +1}{\underset{i=j}{\sum }}x_{i}, \end{array}$$
  where 1 ≤ *j* ≤ *N* − *τ* and *i* = 1,2,3,…, *N*; Figure 1 shows the schematic to obtain the nearest-neighbor moving-window-averaging approach to obtain the new time series.
(2) Template vectors **y**_*k*_^*m*^(*δ*) with dimension *m* and delay *δ* are constructed from z*^τ^* (see Figure 1) at each specific *τ* as the following:
  (2) $$\begin{array}{c} y_{k}^{m}\left(\delta \right)=\left\{z_{k}\;z_{k}+\delta \cdots z_{k}+\left(m-1\right)\delta \right\}, \end{array}$$
  where 1 ≤ *k* ≤ *N* − *mδ*.
(3) The Euclidean distance *d*_*ij*_^*m*^ for each pair of template vectors {**y**_*i*_^*m*^,  **y**_*j*_^*m*^} is calculated using the infinity norm as below:
  (3) $$\begin{array}{c} d_{ij}^{m}\left(\delta \right)={\left\|y_{i}^{m}\left(\delta \right)-y_{j}^{m}\left(\delta \right)\right\|}_{\infty }, \end{array}$$
  where 1 ≤ *i*, *j* ≤ *N* − *mδ* and *j* > *i* + *δ*.
(4) Matched template vector pairs {**y**_*i*_^*m*^,  **y**_*j*_^*m*^} are computed based on a predefined tolerance threshold *r* as
  (4) $$\begin{array}{c} d_{ij}^{m}\left(\delta \right)\leq r. \end{array}$$

In this manuscript, the value for *r* is chosen to be 0.2 times the standard deviation of the raw time series *x*. The delay factor *δ* is chosen to be 1. The total number of matched template vectors is computed and denoted by *n* (*m*, *δ*, *r*).

Steps 2–4 are then repeated for *m* + 1 dimension, and the total number of matched template vectors being computed is denoted by *n* (*m* + 1, *δ*, *r*).

Finally, the improved MSE is calculated as the following:

(5) $$\begin{array}{c} MSE\left(x,m,\delta ,r\right)=-\ln\frac{n\left(m+1,\delta ,r\right)}{n\left(m,\delta ,r\right)}. \end{array}$$

## 3. Materials and Methods

### 3.1. Noise Analysis

We evaluated the performance of the improved MSE technique and compared it with the performance of SE approach with respect to the most common sources of noise: (i) zero mean white noise, (ii) pink noise which has the inverse frequency response (1/*f*), and (iii) brown noise which has the inverse frequency squared response (1/*f*^2^) [26, 27].

White, pink, and brown noises were simulated in MATLAB™, with 10,000 sample points. Ten short time series (TS) versions of these data were created with 250, 500, 750, 1000, 2000, 4000, 5000, 6000, 8000, and 10,000 samples. MSE was calculated via (5) for each noise using different time-scale factors “*τ*” from 1 to 20 over varying time series lengths. Normalized MSE (for *τ* = 1, 2, 3, 5) and SE were calculated by dividing the MSE (and SE) values by the maximum value of MSE (and SE) across varying time series. MSE and SE results for *τ* > 5 are quantitatively similar to that of *τ* = 5 and therefore are not shown.

### 3.2. Description of Datasets for Noise Analysis

To test the robustness of an improved MSE technique in the presence of various noises, we used (1) simplified non-physiological sinusoidal wave and (2) physiological ECG signal, which is the most commonly used time series signal for the diagnostic of various diseases of the heart.

1. A sinusoidal wave with single frequency of 10 Hz and a multifrequency sinusoidal wave with superposition of 2, 5, 10, 15, and 20 Hz frequencies were used. Ten short time series versions of the data were simulated in MATLAB.
2. Noise-free flat baseline ECG was obtained using an electronic ECG simulator with 10,000 sample points at 250 Hz sampling rate. Ten short time series versions of these data were created.

White, pink, and brown noises were added to the noise-free signals and the analysis was performed as described in sub-Section A to compare the performance of MSE and SE techniques.

### 3.3. NSR and AF ECG Discrimination Analysis

Publically available ECG datasets were obtained from the MIT-BIH Physionet database during NSR and AF [28]. Ten NSR and AF datasets of 10-second duration and 250 Hz sampling rate were used for analysis. The signals were not preprocessed for noise removal and *τ* = 3 for MSE calculation. NSR and AF datasets were compared using custom MATLAB software. Mann–Whitney test with *p* value of 0.01 was used for testing statistical significance and was performed using OriginPro software (OriginLab Corporation, Northampton, Massachusetts).

### 3.4. Optical Mapping Data from Isolated Rabbit Hearts

Optical mapping movies during a single rotor or figure-of-8 reentry were obtained from an isolated rabbit heart by inducing ventricular tachycardia via burst pacing as described previously [29, 30]. The movies were 3-second long, acquired at 600 frames per second temporal and 64 × 64-pixel spatial resolution. Two-dimensional (2D) MSE maps were generated for both single rotor and figure-of-8 reentry using the MSE values with the scale factors *τ* = 1, 2, and 3 at each pixel location across all the frames. For comparison purposes, the 2D SE map was computed. A custom MATLAB (MathWorks Inc., Natick, MA) program was developed for all processing. Supplemental videos SV1 and SV2 are provided for reference that shows the phase movie of single and double rotor, respectively.

## 4. Results

### 4.1. Noise Analysis

**Figure 2:**
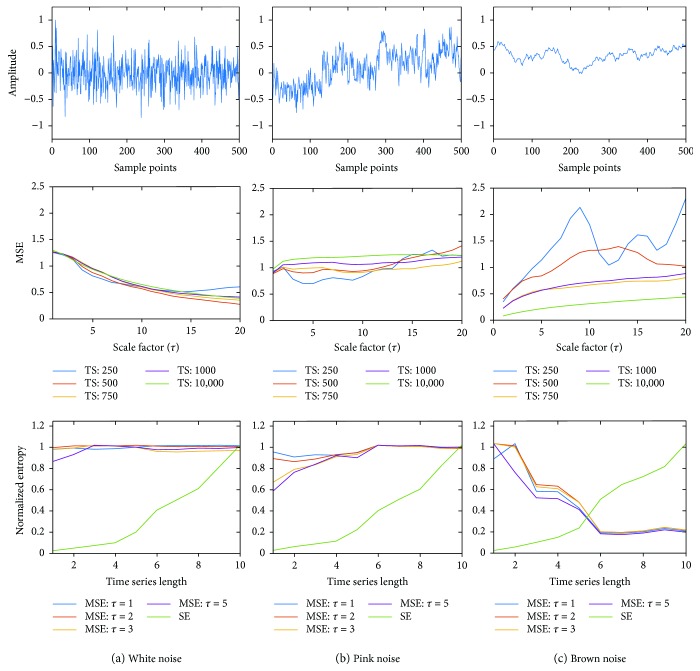
(a) Top panel shows white noise with 500 sample points; Middle panel shows the MSE for various time series (TS) lengths; bottom panel shows normalized MSE (for scale factors *τ* = 1,2,3, and 5) and SE; (b) top panel shows pink noise with 500 sample points; middle panel shows the MSE for various time series (TS) lengths; bottom panel shows normalized MSE (for scale factors *τ* = 1,2,3, and 5) and SE; (c) top panel shows brown noise with 500 sample points; middle panel shows the MSE for various time series (TS) lengths; bottom panel shows normalized MSE (for scale factors *τ* = 1,2,3, and 5) and SE.

Figure 2 shows the robustness of MSE and SE techniques with respect to different types of noise: white (a), pink (b), and brown (c). The middle row of Figures 2(a)–2(c) shows the MSE values as a function of *τ* for varying TS lengths. As expected, for white noise, MSE monotonically decreases as *τ* increases, and changing TS length does not affect the data. For pink noise, MSE increases with the increase of the TS length, and for long TS (1000 samples), MSE does not depend on *τ*. For brown noise, MSE decreases with the increase in the TS length and does not depend on *τ* for long TS. These results demonstrate the robustness of MSE since the expected behavior is observed for each noise. The bottom row of Figures 2(a)–2(c) shows the normalized values of MSE (for different *τ*) and SE as a function of the TS length. As seen from these data, the values of SE decrease as TS decreases for all types of noises, while MSE values do not depend on the TS length. These results demonstrate that the performance of MSE is better than SE, especially for short time series.

The MSE of white noise is expected to show a monotonically decreasing response with higher scale factors [14–16] which was seen in Figure 2(a) middle panel with increasing scale factor due to the nearest neighbor averaging that leads to lower MSE for white noise is shown. For pink noise which has a 1/*f* response, higher MSE than white noise is expected but with a constant value across multiple time scales [14]. As expected, MSE levels out at higher time series lengths above 1000 sample points across the different time scales seen in Figure 2(b) middle panel. This means that for a sampling rate of 250 Hz, MSE can capture the complexity with just 4 s of data. Similarly, for brown noise, MSE is expected to be constant and as seen from Figure 2(c) middle panel after a TS length of 750 sample points, MSE is more or less the same across multiple time scales. Figures 2(a)–2(c) bottom panel demonstrates the fact that SE estimates lower values for short time series and gradually increases with increasing time series length for all three types of noise. MSE has higher values even for the shortest time series, thereby capturing the complexity better than SE. Overall, the results indicate that if at least 1000 sample points are available, MSE can capture the complexity robustly compared to SE. For most physiological monitoring, 250 Hz sampling frequency is common, which indicates that 4 s short time series data should be sufficient for robust analysis using MSE.

**Figure 3:**
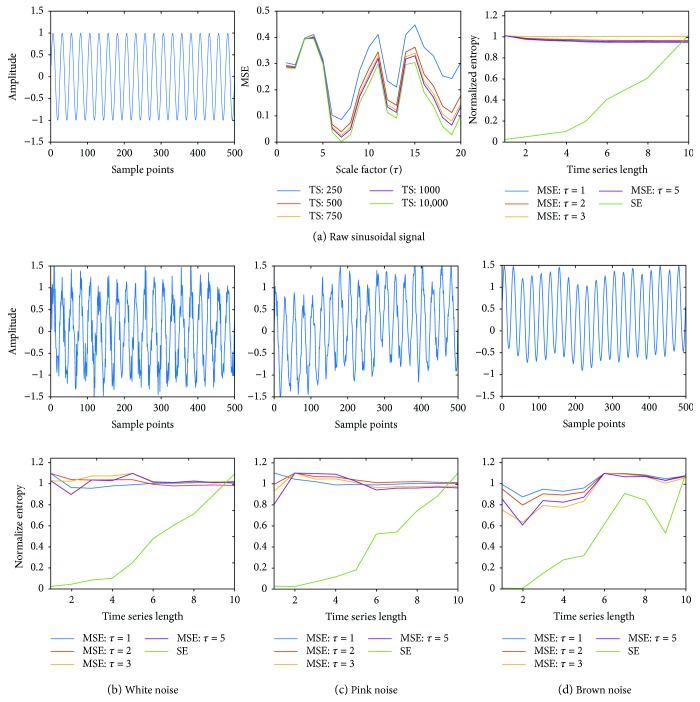
(a) Left panel shows single-frequency (10 Hz) sinusoidal wave with 500 sample points; middle panel shows the MSE for various length time series across several scaling factors; right panel shows normalized MSE (for scale factors *τ* = 1–10) and SE; (b) top row shows sinusoidal wave with white noise, (c) pink noise, and (d) brown noise; (b) bottom row shows normalized MSE (for scale factors *τ* = 1–10) and SE for sine wave with white noise, (c) pink noise, and (d) brown noise, respectively.

Figure 3 demonstrates the robustness of MSE compared with SE technique for single-frequency sinusoidal wave in the absence and presence of different noises. Figure 3(a) shows the single-frequency sinusoidal wave at 10 Hz for 500 sample points (left panel), the MSE values as a function of *τ* for varying TS (middle panel), and the normalized values of MSE (for different *τ*) and SE as a function of TS length (right panel) in the absence of noise. These data demonstrate higher efficacy of the MSE technique in capturing the complexity of the sine wave than that of SE, which only works well for larger TS lengths. The robustness of the MSE and SE techniques in identifying the complexity of a single-frequency sinusoidal wave in the presence of noise is shown in Figure 3 for the white (b), pink (c), and brown (d) noises. The top row of Figures 3(b)–3(d) shows the amplitude of sinusoidal wave with noise, while the bottom row shows the normalized values of MSE (for different *τ*) and SE as a function of TS length. Our results suggest that MSE captures the complexity of sinusoidal waves better than SE in the presence of these noises.

**Figure 4:**
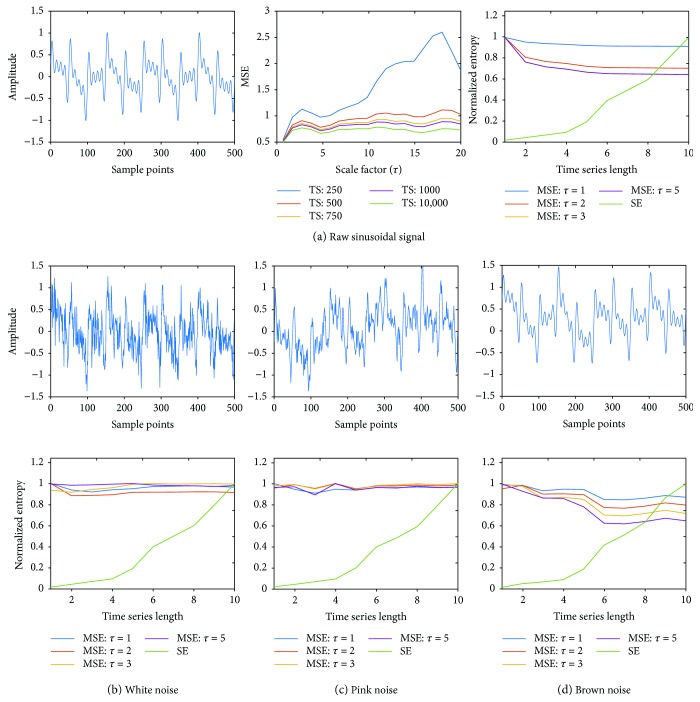
(a) Left panel shows multifrequency sinusoidal wave with 500 sample points; middle panel shows the MSE for various length time series across several scaling factors; right panel shows normalized MSE (for scale factors *τ* = 1–10) and SE; (b) top row shows sinusoidal wave with white noise, (c) pink noise, and (d) brown noise; (b) bottom row shows normalized MSE (for scale factors *τ* = 1–10) and SE for sinusoidal wave with white noise, (c) pink noise, and (d) brown noise, respectively.

**Figure 5:**
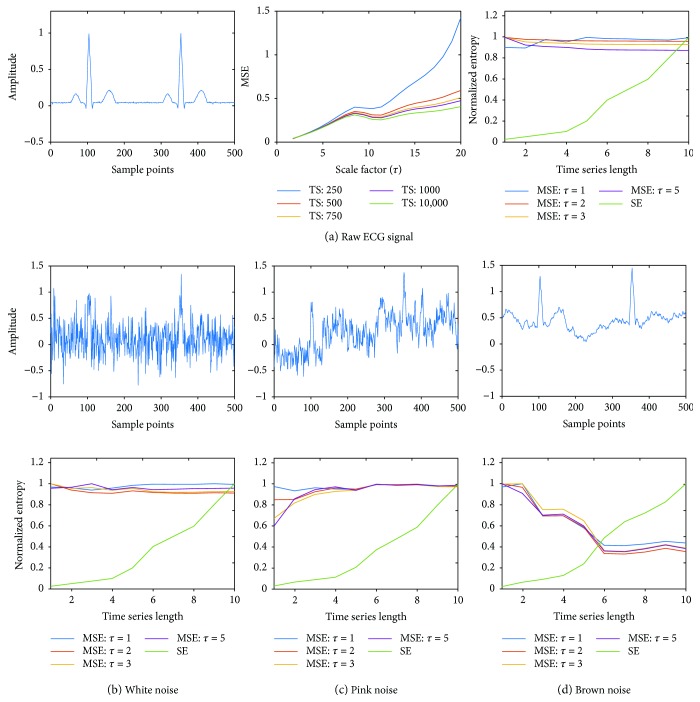
(a) Left panel shows sample ECG trace with 500 sample points; middle panel shows the MSE for various length time series across several scaling factors; right panel shows normalized MSE (for scale factors *τ* = 1–10) and SE; (b) top row shows sinusoidal wave with white noise, (c) pink noise, and (d) brown noise; (b) bottom row shows normalized MSE (for scale factors *τ* = 1–10) and SE for sinusoidal wave with white noise, (c) pink noise, and (d) brown noise, respectively.

Figures 4 and 5 show the results for the multifrequency sinusoidal wave and the noise-free flat ECG, respectively. Similar to the response seen in Figure 2 for raw noise, (b)–(d) of Figures 4 and 5 demonstrate that SE is very small for short TS and gradually increases with increasing TS length, while MSE has high values even for the shortest TS, thereby capturing the complexity better than SE. The results demonstrate the efficacy of the novel MSE technique in quantifying the complexity of complex time series data in the presence of noise better than that of the commonly used SE approach.

### 4.2. ECG Analysis

**Figure 6:**
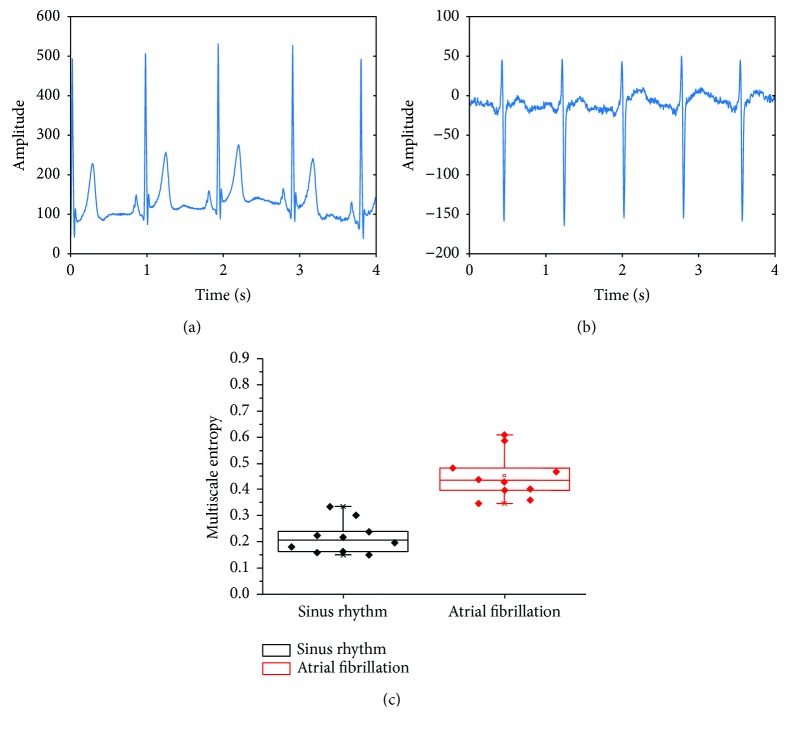
(a) Representative example of an ECG trace with normal sinus rhythm; (b) representative example of an ECG trace with AF; (c) box plot showing MSE values for sinus rhythm and AF ECG datasets. The NSR and AF were significantly different (*p* < 0.01) on MSE.

Figure 6 shows the raw ECG with NSR (a) and AF (b). Note that visual inspection of these traces cannot be used to correctly discriminate between NSR and AF.  Figure 6(c) shows the boxplot of MSE values for 10 AF and NSR datasets demonstrating statistically significant differences (*p* < 0.01) and therefore accurate discrimination between NSR and AF. As observed in Figures 6(a) and 6(b) visually, it is difficult to interpret the difference between NSR and AF on the ECG as the chaotic nature of AF manifests itself into small morphological disturbances which need robust algorithms to effectively capture the complexity. MSE robustly discriminates NSR and AF.

### 4.3. Identification of Pivot Point of the Rotor

**Figure 7:**
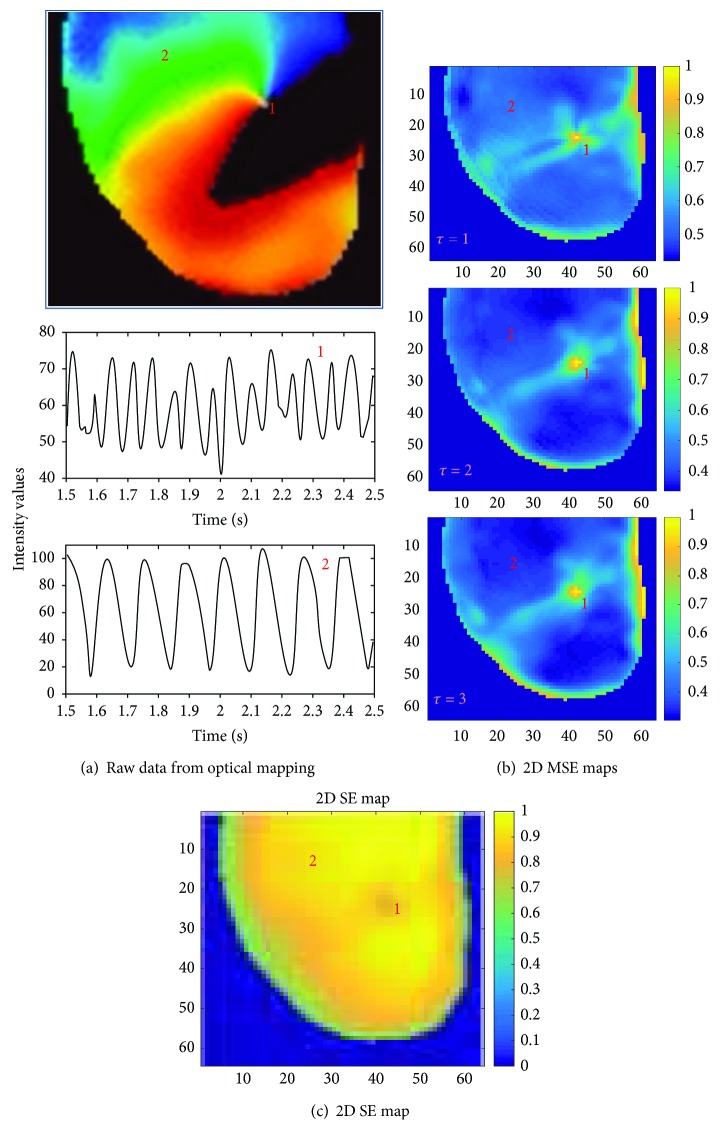
(a) Top panel shows a representative example of a single rotor. Pixel locations “1” represent rotor core region and “2” represent rotor periphery; bottom panel shows corresponding voltage traces at those pixel locations. (b) Normalized 2D MSE maps; top panel with scale factor *τ* = 1; middle panel with *τ* = 2; bottom panel with *τ* = 3, correctly identifying rotor core regions; (c) normalized 2D SE map with lower SE values at the rotor core.

A snapshot of a phase movie of a single rotor in isolated rabbit heart is shown in Figure 7(a). In this movie, different colors represent different phases of the action potential, and the pivot point of the rotor can be easily identified as the point where different phases converge. Corresponding voltage traces from the core (pixel “1”) and periphery of the rotor (pixel “2”) are also shown. At the core of the rotor, broader distribution of voltage amplitude occurs due to the chaotic nature at the rotor pivot point and therefore, a higher MSE value was expected. At the periphery of the rotor, more uniform electrical activity is observed and hence, a lower MSE value was expected.  Figure 7(b) shows the 2D MSE maps for three time-scale factor *τ* = 1, 2, and 3. Note the MSE technique can accurately identify the location of the pivot point of the rotor for each *τ*. As seen from (b), the pivot point has higher MSE values than the periphery thereby enabling its precise localization, and higher values of “τ” results in better contrast between the rotor core and periphery.  Figure 7(c) shows the normalized 2D SE map of the same single rotor. It is important to note that although SE can correctly identify the pivot point of the rotor, the contrast between SE values at the core and the periphery is low, which challenges accurate identification.

**Figure 8:**
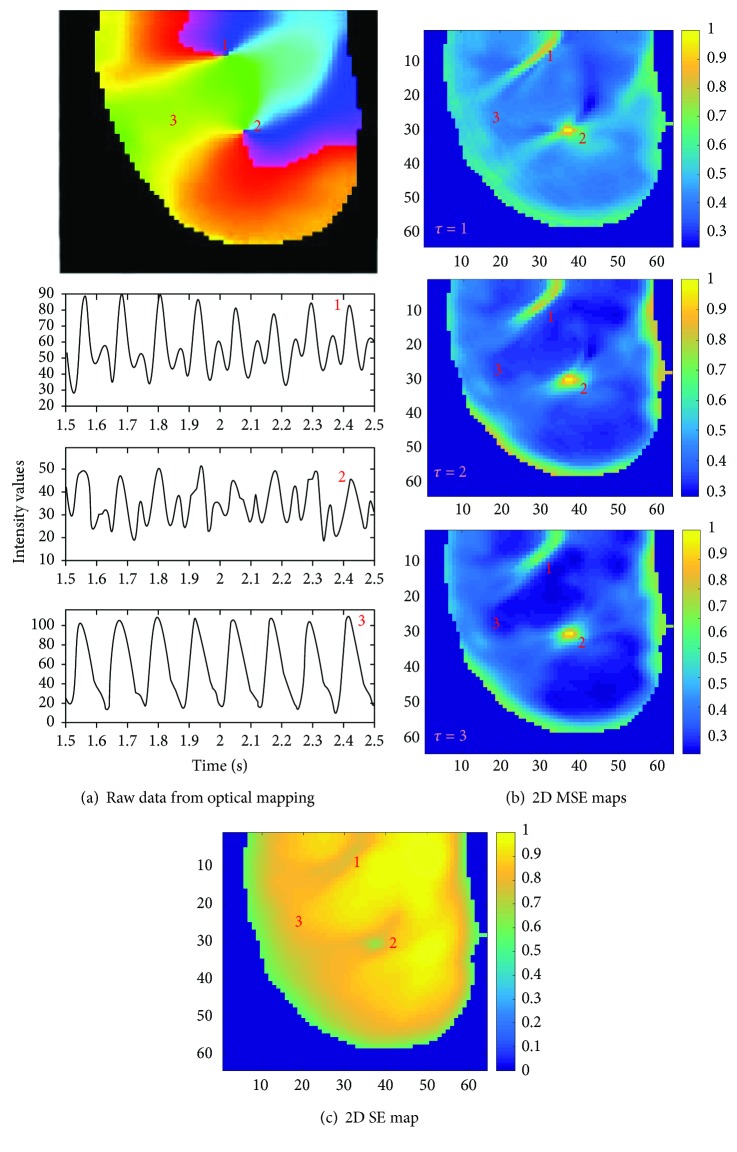
(a) Top panel shows a representative example of a figure-of-8 reentry. Pixel locations “1” and “2” represent rotor core region and “3” represent rotor periphery; bottom panel shows corresponding voltage traces at those pixel locations. (b) Normalized 2D MSE maps; top panel with scale factor *τ* = 1; middle panel with *τ* = 2; bottom panel with *τ* = 3, correctly identifying rotor core regions; (c) normalized 2D SE map with lower SE values at the rotor core.

Figure 8(a) shows a snapshot of a phase movie for an example of figure-of-8 reentry in an isolated rabbit heart. Similar to Figure 7, one can see that the MSE technique can correctly identify the location of the pivot points of the rotors for each *τ* and that the performance of the MSE technique is much better than SE observed in Figures 8(b)–8(c).

As seen in Figure 7(b), it is seen that a scale factor of *τ* = 1 was sufficient enough to provide the necessary contrast to identify the rotor pivot points with higher MSE values at the rotor pivot point than that in the periphery. Higher scale factor values provided improved contrast as seen when comparing 2D MSE maps in Figure 7(b). Similar results are observed for figure-of-8 reentry data seen in Figure 8. It is interesting to note that at pixel location “1,” the rotor meanders to some extent which is also captured robustly by MSE compared to SE.

## 5. Discussion

In this study, we developed an improved MSE technique with nearest-neighbor moving-average kernel and demonstrated that it can be successfully used for the analysis of nonlinear and nonstationary short time series physiological data. The MSE robustly estimated the complexity of short time series data compared to SE with various noises such as white, pink, and brown noises. Major findings of this manuscript are the following: (1) MSE discriminated NSR and AF on single-lead ECG of 10 s recordings without any preprocessing steps and (2) MSE precisely identified the pivot point of the rotor (single and figure-of-8 reentry) with 3 s optical mapping data from isolated rabbit hearts by providing better contrast between the rotor core and the periphery region when compared to the SE approach. The efficacy of MSE technique was clearly demonstrated with short time series analysis which can be used in a variety of other physiological applications.

### 5.1. Sinusoidal Wave Analysis

Sinusoidal wave analysis is the most elegant approach to demonstrate the efficacy of the improved MSE technique over the conventionally used SE approach for short time series analysis of biomedical signals. We demonstrated that both for single-frequency and multifrequency sinusoidal waves with added noise, SE underestimated the complexity at short time series for all three noise cases and performed better at longer time series lengths. However, MSE was robust even at shorter time series with 1000 sample points in the presence of the three types of noise. The results suggest the value of MSE technique in analyzing complex short time series physiological signals that can be contaminated with these noises and its use for the prognosis and diagnosis of various disease states.

### 5.2. Noise-Free ECG Analysis

ECG analysis is very commonly used for a wide variety of cardiac conditions to yield information regarding the state of the heart. Since most remote and ambulatory real-time ECG monitoring present at most 3–5 seconds of ECG data, conventional complexity analysis methods such as SE are limited. However, we demonstrated that MSE robustly estimated the complexity of short time series ECG data even in the presence of noise.

### 5.3. Discrimination between NSR and AF

AF is the most common sustained cardiac arrhythmia that is associated with increased risk of stroke, heart failure, and death affecting more than 2.3 million people in the United States and over 30 million people worldwide [31]. Although the persistent form of AF can be detected relatively easy, detecting paroxysmal AF is often a challenge since continuous monitoring is required, which in turn requires methods to discriminate NSR from AF through large quantities of data [32].

Although there are several methods available for NSR and AF discrimination, they face limitations in successfully detecting AF with high sensitivity and specificity using short-time ECG data [32–34]. The major issues with these approaches are that they often distort the ECG by several preprocessing steps with filters, they do not provide reliable discrimination using short ECG time series data, and many of them lack real-time capability that makes it difficult to trust the data for diagnosis and treatment. Here, we demonstrated that the improved MSE technique can robustly discriminate AF from NSR using a single-lead ECG. The results motivate the application and use of this MSE technique for many hand-held and remote ECG monitors to autodetect AF.

### 5.4. Identification of Pivot Points of Rotors

Catheter ablation to treat paroxysmal AF has been shown to be up to 87% successful using pulmonary vein (PV) isolation [35–40]. However, in patients with persistent AF ablation, it is challenging since the location of the triggers is unclear, and it has been shown that triggers commonly arise outside the PVs. Recent research suggests that AF ablation has a success rate of 28% with 51% after multiple repeat procedures in persistent AF [41].

It is believed that rotors are caused by reentrant mechanisms which might be responsible for maintaining persistent AF. Identification of the rotor pivot point as a suitable ablation target has been the research focus for many investigators. However, these investigations are challenged with short time series data in the clinical setting. Here, we used optical mapping data in which rotors can be clearly visualized, and we demonstrated that the improved MSE technique can precisely identify pivot points in both single rotor and figure-of-8 reentry, thus offering a robust mapping tool to guide identification of AF ablation targets. In the clinical setting, electrogram recordings are frequently limited to 2.5–5-second segments due to the need for frequent catheter repositioning during the procedure, challenging conventional mapping approaches to precisely identifying substrates in AF and other arrhythmias.

### 5.5. Limitations

A limitation of the improved MSE technique is the need to select a correct choice of the time scale factor “*τ*.” Since the nearest-neighbor moving averaging is employed, large time scales will cause excessive smoothing of the data which may lead to loss of some complexity information. Therefore, caution should be used in the appropriate choice of scaling factor. The results from this study suggest that a scale factor of *τ* = 3 may be a reasonable starting point for many applications, but clinical validation is needed.

In addition, our analysis was limited to relatively small number of datasets. More rigorous evaluation using a larger number of datasets is critical in order to validate these findings for ECG discrimination as well as for rotor identification. Finally, we did not specifically evaluate ex vivo examples of AF but only of more organized cardiac arrhythmias to determine critical rotor elements. Given the higher-order complexity associated with AF, further study is needed in experimental models of AF to validate the use of MSE for characterization of rotors in these arrhythmia examples.

## 6. Conclusions

An improved MSE technique with nearest-neighbor moving-average kernel was developed to eliminate the systematic bias from one-sided averaging. The results demonstrate that MSE technique can be successfully used for the analysis of nonlinear and nonstationary short time series physiological data. Compared to the commonly used SE approach, MSE robustly estimated complexity with short time series data with various noises such as white, pink, and brown noises. The MSE discriminated NSR and AF on single-lead ECG of 10 s recordings without any preprocessing steps and precisely identified the pivot point of the rotors with 3 s optical mapping data from isolated rabbit hearts by providing better contrast between the rotor core and the periphery region when compared to the SE approach. Wide-range application of this technique on a variety of time series data can open new avenues for analysis and interpretation.

## 7. Future Work

Future work will focus on further validating the efficacy of NSR and AF discrimination on a larger dataset. Also, the MSE algorithm will be validated with a variety of rotor data for accurate identification of ablation targets using both optical mapping and intracardiac electrograms that can guide patient-specific mapping and ablation.

## Supplementary Materials

Supplementary 1 SV1: phase movie of the single rotor from optical mapping of isolated rabbit heart.

Supplementary 2 SV2: phase movie of the figure-of-8 reentry from optical mapping of isolated rabbit heart.
